# Improvement of plant growth and seed yield in *Jatropha curcas* by a novel nitrogen-fixing root associated *Enterobacter* species

**DOI:** 10.1186/1754-6834-6-140

**Published:** 2013-10-01

**Authors:** Munusamy Madhaiyan, Ni Peng, Ngoh Si Te, Cheng Hsin I, Cai Lin, Fu Lin, Chalapathy Reddy, Hong Yan, Lianghui Ji

**Affiliations:** 1 Biomaterials and Biocatalysts Group, Temasek Life Sciences Laboratory, 1 Research Link, National University of Singapore, Singapore 117604, Republic of Singapore; 2 Joil Pte Ltd, 1 Research Link National University of Singapore, Singapore 117604, Republic of Singapore

**Keywords:** Bacterial endophytes, Nitrogen fixation, *Enterobacter*, Plant growth promotion, *Jatropha curcas* L, Biofuel

## Abstract

**Background:**

*Jatropha curcas* L. is an oil seed producing non-leguminous tropical shrub that has good potential to be a fuel plant that can be cultivated on marginal land. Due to the low nutrient content of the targeted plantation area, the requirement for fertilizer is expected to be higher than other plants. This factor severely affects the commercial viability of *J. curcas*.

**Results:**

We explored the feasibility to use endophytic nitrogen-fixing bacteria that are native to *J. curcas* to improve plant growth, biomass and seed productivity. We demonstrated that a novel N-fixing endophyte, *Enterobacter* sp. R4-368, was able to colonize in root and stem tissues and significantly promoted early plant growth and seed productivity of *J. curcas* in sterilized and non-sterilized soil. Inoculation of young seedling led to an approximately 57.2% increase in seedling vigour over a six week period. At 90 days after planting, inoculated plants showed an average increase of 25.3%, 77.7%, 27.5%, 45.8% in plant height, leaf number, chlorophyll content and stem volume, respectively. Notably, inoculation of the strain led to a 49.0% increase in the average seed number per plant and 20% increase in the average single seed weight when plants were maintained for 1.5 years in non-sterilized soil in pots in the open air. *Enterobacter* sp. R4-368 cells were able to colonize root tissues and moved systemically to stem tissues. However, no bacteria were found in leaves. Promotion of plant growth and leaf nitrogen content by the strain was partially lost in *nifH*, *nifD*, *nifK* knockout mutants, suggesting the presence of other growth promoting factors that are associated with this bacterium strain.

**Conclusion:**

Our results showed that *Enterobacter* sp. R4-368 significantly promoted growth and seed yield of *J. curcas.* The application of the strains is likely to significantly improve the commercial viability of *J. curcas* due to the reduced fertilizer cost and improved oil yield.

## Background

The rapid rise of fossil fuel price, diminishing global fuel reserve and the concerns about global warming, resulted from the accumulation of atmospheric Greenhouse Gases, have served as the three catalysts in the recent biofuel boom [1]. The environmental benefit of biofuel consumption is attributed from the widely believed outcome of reduced CO_2_ emission. Recently, a new issue has been brought up for debate regarding the benefit of biofuels. Nitrous oxide (N_2_O) is produced naturally in the soil during the microbial processes of nitrification and denitrification. A significant percentage of the nitrogen fertilizer used in biofuel production is converted to reactive nitrogen N_2_O, a greenhouse gas which has 310 times the ability of CO_2_ to trap heat in the atmosphere [2]. The benefit of CO_2_ mitigation through biofuel consumption will be diminished if the use of nitrogen fertilizer is not controlled [3-5].

*Jatropha curcas* (Jatropha) is a small woody plant belonging to the *Euphorbiaceae* family. Several unique characteristics of Jatropha make it an ideal plant for biodiesel production [6-8]. These include the ability to grow on marginal land, low requirement for water, a non-food crop status and fast oil production in 0.5-2 years after planting compared to more than 3 years for oil palm. Accordingly, several Asian countries, particularly Indonesia and India, have made ambitious plans to promote Jatropha plantation. Apart from breeding programs for high yielding Jatropha varieties [9-11], agronomical practices, such as the application of inorganic fertilizer [12] and plant growth regulators, have also been reported to improve seed yield [13,14]. Application of nitrogen fertilizer has become an essential practice in modern agriculture, as it is vital to maintain competitive crop productivity. It is well known that legumes require much less input of nitrogen fertilizer owing to their natural capability to fix atmospheric nitrogen. This is attributed to nitrogen-fixing rhizobia (diazotrophs), mostly belonging to species in the *Rhizobium*, *Sinorhizobium*, *Mesorhizobium* and *Bradyrhizobium* genera [15-18], forming a mutually beneficial symbiotic relationship with the bacteria supplying nitrogen source to the plants while drawing carbon source from the plants cells [19-21]. The formation of symbiotic nitrogen-fixing nodules involves complex genetic and chemical interactions between the diazotroph and the host. For example, the symbiosis between *Sinorhizobium meliloti* and its plant hosts begins when the plant secretes an array of betaines and flavonoids into the rhizosphere. These compounds attract *S. meliloti* to the surface of the root hairs of the plant where the bacteria begin secreting nodulation factor [22,23]. As a consequence, symbiotic nodule-forming nitrogen fixation is found almost exclusively in legume species [24].

Interestingly, an increasingly number of diazotrophic species, e.g., *Azospirillum*, *Herbaspirillum*, *Burkholderia*, *Gluconacetobacter*, have been reported to form atypical symbiotic relationship with plants [25-27]. They often grow on the surface of the root system (rhizobacteria) although some are able to infect plant tissues (endophytic bacteria) and perform nitrogen fixation, which in turn promotes plant growth [28]. Perhaps, the best examples of endophytic nitrogen-fixation can be found in sugarcane and wild rice, with *Herbaspirillum, Gluconacetobacter, Enterobacter, Azospirillum, Swaminathania* and *Acetobacter* being the major contributors of nitrogen-fixing species [27,29-32]. Photosynthetic Bradyrhizobia are the natural endophytes of the African wild rice *Oryza breviligulata* while the intercellular colonization and growth-promoting effects of a *Methylobacterium* species was observed in common rice although the latter was believed to result from phytohormone secretion by the bacterium [33,34].

As Jatropha is targeted to be grown in marginal land where soil nutrient is low, the requirement for nitrogen fertilizer will be higher than other crops. Therefore, any technology that reduces nitrogen fertilizer usage will be highly desirable [6-8]. To date, there is little research on naturally occurring diazotrophs in neither this plant nor the application of these microbes for improvement of plant productivity. Here we present our investigation on the isolation, characterization and application of root associated nitrogen-fixing bacteria from Jatropha cultivars*.*

## Results

### Isolation and characterization endophytic nitrogen-fixing bacteria from Jatropha

**Figure 1:**
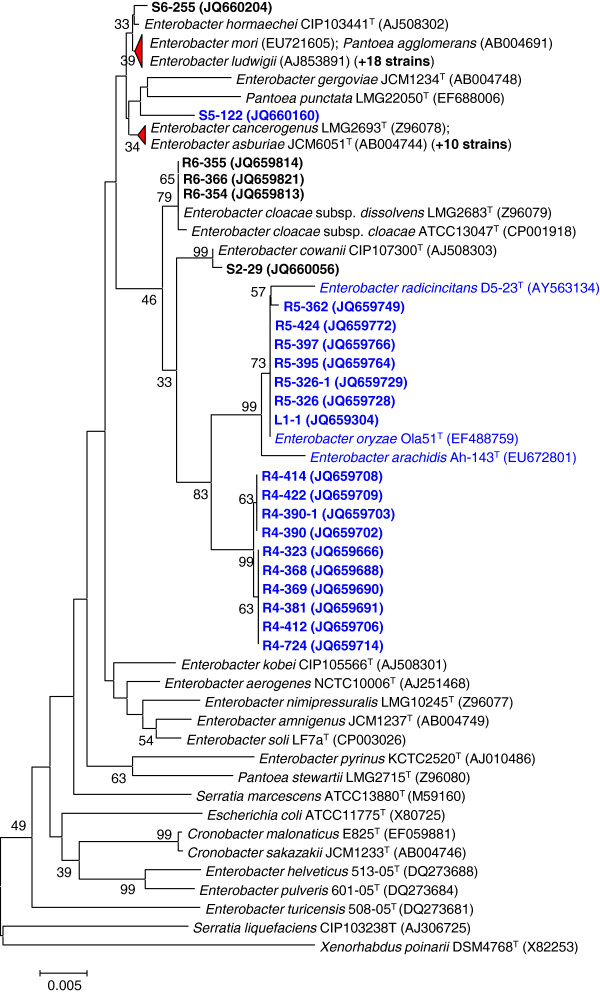
**Taxonomical classification of isolated *Enterobacter *strains.** Phylogenetic tree was based on 16S rRNA gene sequences, of which the Genebank accession numbers are shown in the brackets. Bootstrap values (expressed as percentages of 1000 replications) greater than 30% are shown at the branch points. Bar: 0.005 substitutions per nucleotide position. Potential nitrogen-fixing strains, which were positive for the *nifH* gene as judged by PCR amplifications and sequence confirmation, are shown in indicated in blue. The number of isolates highly related to *E. ludwigii* and *E. asburiae* are indicated by the number in the bracket, respectively.

**Figure 2:**
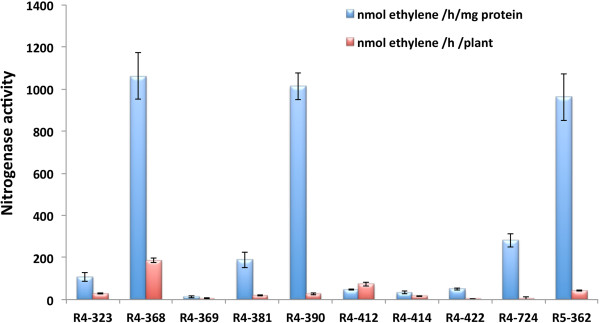
**Nitrogenase activities of selected isolates from Jatropha cultivars.** Acetylene reduction (AR) activity was measured *in vitro* (pure cultures) and *in planta* conditions. Error bars represent SD (n = 4).

Endophytic nitrogen (N)-fixing bacteria were isolated from tissues of three germplasm accessions of Jatropha based on their ability to grow in nitrogen-free medium. Analysis of 16S rRNA sequences of candidate strains isolated revealed that 54 of them were closely related to known species in the *Enterobacter* genus. Six strains are clustered with the known species, such as *Enterobacter radicincitans* D5-23^T^ and *Enterobacter oryzae* Ola51^T^, whereas the other 10 strains formed a distinct new cluster, which are characterized in detail in this report (Figure 1). After confirmation of the presence of *nifH* genes by PCR and DNA sequencing, we selected a few *Enterobacter* strains and examined their ability to fix atmospheric nitrogen under *in vitro* and *in planta* conditions by acetylene reduction (AR) assay (nitrogenase assay). The *Enterobacter* strains that showed high AR activity *in vitro* were selected for the assessment of nitrogenase activity *in planta* by inoculation to Jatropha seedlings. The AR-activity *in vitro* varied widely among the strains, ranged from 12.4 to 1060.7 nmol C_2_H_4_ mg protein^–1^ h^–1^ while the *in planta* AR activity ranged from 43.7 to 186.3 nmol C_2_H_4_ plant^-1^ h^-1^ (Figure 2). One of the strains, R4-368 showed the best AR activity, and was thus selected for further studies.

### Inoculation of *Enterobacter* sp. strain R4-368 improved production of biomass and seeds

**Figure 3:**
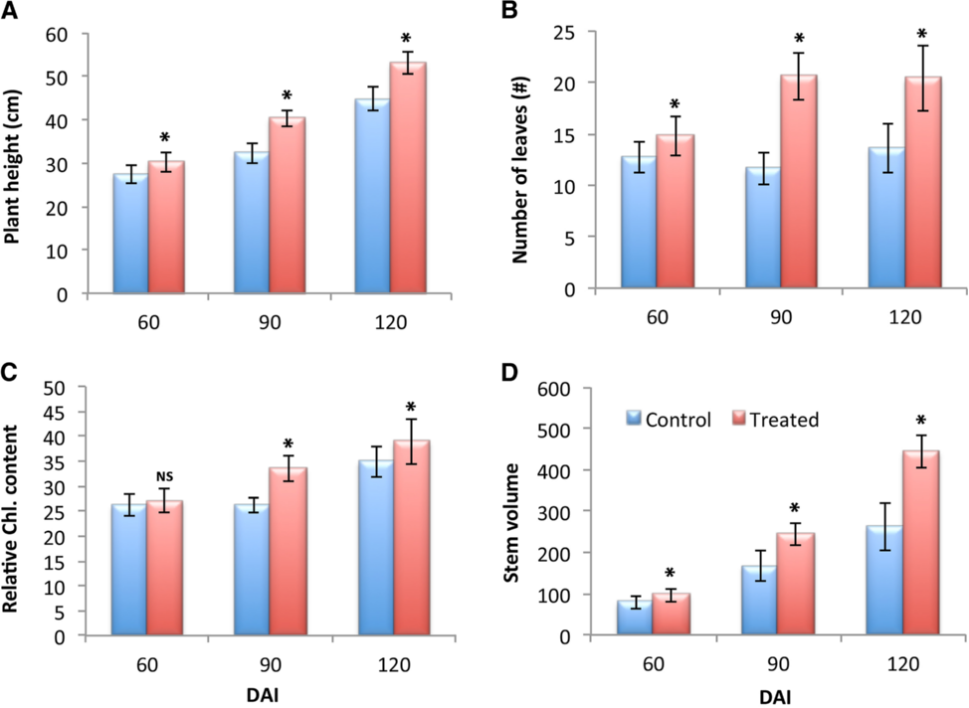
**Growth promotion of*Jatropha curcas*by R4-368.** Seedlings were inoculated with R4-368 and grown germination in trays and then transferred to pot with one seedling/pot with unsterilized garden soil and grown in open air for 1.5 years. Plant height **(A)**, number of leaves **(B)**, relative chlorophyll content **(C)** and stem volume **(D)** were recorded once in 30 days. * means significant difference at 5% threshold between treated and control using DMRT. NS-not significant.

**Figure 4:**
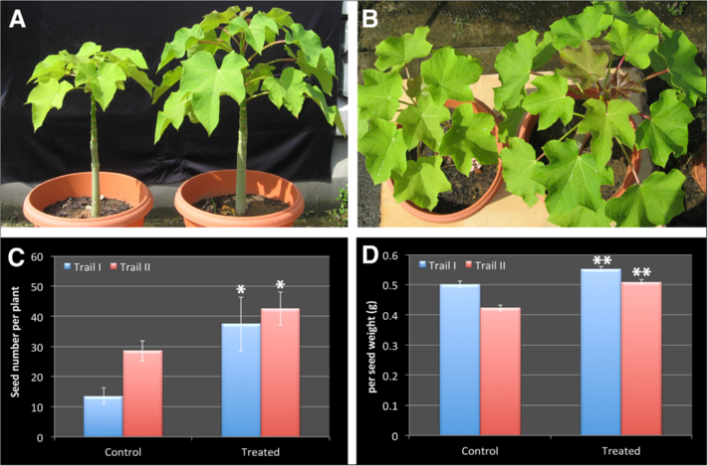
**Effect of *Enterobacte r*R4-368 inoculation on seed production.** Plants were planted in pots in sterilized soil (compost/sand mix at 1:1 ratio and in ɸ23 cm, 18 cm height pots; named as Trial I) or none-sterilized soil (nutrient poor clay soil in ɸ30 cm, 28 cm height pots; named as Trial II). Trial I and Trial II were maintained in different locations and started in different seasons. R4-368 cell suspension (1.2 OD_600nm_, 50 ml/pot) was applied to the plant root system at 21 days after seed germination. Fertiliser was applied regularly at about half of the recommended dose of approximately 50:30:30 g/plant/year. **(A)** Plant height **(B)** leaf canopy. Seed set numbers per plant **(C)** (n = 8 in Trial I and n = 12 in Trial II) were measured at 480 and 520 DAI in Trail I and Trail II respectively and single seed weight **(D)** was calculated based the average of 180 randomly selected seeds per treatment except the non-treated for Trial I where only 117 seeds were measured. Error bars indicate standard error. * and ** indicate statistically significant between the treated and non-treated populations (*P < 0.05 for seed set per plant and **P < 0.01 for single seed weight) by Student’s t-test.

To confirm that *Enterobacter* sp. R4-368 was able to improve agronomic traits of Jatropha plant, its seedlings were inoculated with the strains by seed soaking. As expected, at 45 days after sowings [35], the inoculated plants accumulated 30.51% higher dry biomass associated with significantly increased leaf chlorophyll content and growth vigour than the non-inoculated control plants (Table 1). To further establish the growth-promoting effect of R4-368, Jatropha plants were inoculated with the strain in the root system by watering a bacterium suspension into the soil that had been sterilized before planting. These plants were maintained in the open air. Again, R4-368 treated plants showed significant improvements in plant height, leaf counts or plant canopy and leaf chlorophyll content compared with the untreated control plants (Figure 3). At 120 DAI, treated plants recorded an increase of 18.3%, 50.3%, 11.4% and 69.5% over the control mock-inoculated plants in plant height, leaf counts, leaf chlorophyll content and stem volume respectively (Figure 3A-D). To evaluate the effects of bacterial inoculation on seed production, plants were grown in sterilized and non-sterilized soil in large pots and maintained in the open air. Notably, the average seed set number per tree was increased by approximately 177% at the end of the observation period when sterilized soil was used. The increase was reduced to approximately 49.0% when non-sterilized soil was used (Figure 4C). Student’s t-test showed that the treated populations produced significantly more seed sets than non-treated ones in both experiments (p < 0.05). The average seed weight was increased approximately 10% in Trial I and 20% in Trial II, both increases being very significant according to Student’s t-test of individual seed weight (p < 0.01). The seed set improvement was mainly attributed to an increase of female–male flower ratio, which led to a corresponding increase of fruit and seed sets. There appeared to have a slight increase (~15.2%) of total flower numbers (Additional file 1: Table S1).

**Table 1:** Effects of R4-368 inoculation on the early growth parameters of Jatropha

**Treatments**^**a**^	**Relative chlorophyll content**	**Nitrogenase activity**^**b**^	**SVI**^**c**^	**RG**^**d**^	**Biomass**^**e**^
Control	34.91 ± 3.91	2.15 ± 0.25	2024.2 ± 33.7	23.50 ± 2.50	117.9 ± 3.67
R4-368	36.13 ± 3.13	8.70 ± 0.25	3182.0 ± 170.5	25.76 ± 2.76	153.9 ± 5.83
LSD (*P ≤ 0.05*)	1.94	1.24	134.2	0.65	14.2

^a^After seed soaking, the seeds (50 seeds/replicate, n = 3) were drained and sown in trays containing pot mixture and then placed in a glass house and maintained at 28°C.

^b^Value expressed in nmol C_2_H_4_ day^-1^ seedlings^-1^. Each value represents mean ± standard deviation (SD) of three replicates per treatment.

^c^Seedling vigour index [36] was calculated using the formula: SVI = % germination × seedling length (shoot length + root length) in cm.

^d^Rate of germination (RG) was calculated using the following formula, RG = ∑ Ni/Di where Ni is the number of germinated seeds in a given time, and Di is the time unit (day).

^e^Each value represents mean of three replicates and expressed in grams. Samples were measured at 45 DAS.

### Characterization of the R4-368 nitrogen-fixation genes

**Figure 5:**
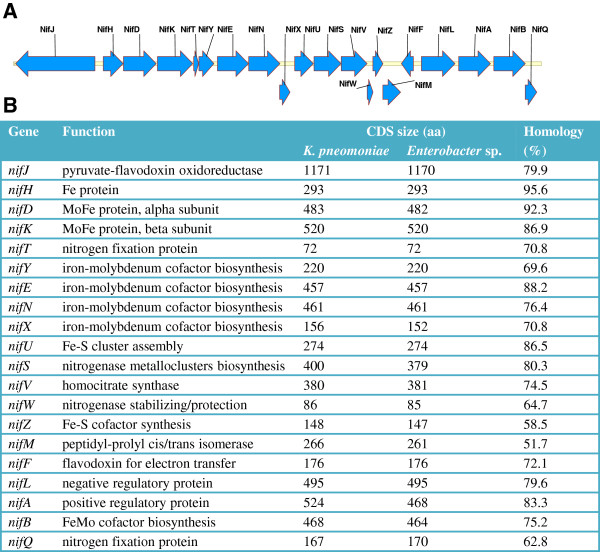
***nif *gene operon in *Enterobacter*sp. R4-368.(A)** Organization of *nif* gene cluster. The coding DNA sequence (CDS) was predicted with RAST [39] and the CDS diagram was generated with Vector NTI v10. **(B)** Comparison of *nif* genes between *Enterabacter* sp. R4-368 and *K. pneumonia* (GenBank: ×13303.1).

A shot-gun sequencing using the 454 sequencing method yielded 76 contigs which are more than 0.5 kb in size. Blast search of the contigs against the nucleotide and microbial genome databases at the NCBI revealed a region of approximately 23 kb which shows high homology to the *nif* operons (GenBank: ×13303.1) of *Klebsiella pneumonia*, one of the best characterized plant root-associated nitrogen fixing bacteria [37,38]. Examination of the annotations, which was done by the RAST (Rapid Annotation using Subsystem Technology) automated annotation service [39], confirmed the presence of a complete *nif* gene cluster with identical organization to those found in *K. pneumonia* (Figure 5A). Alignment of the two *nif* gene clusters revealed an overall sequence identity of 92.5% at the DNA level. The 20 predicted proteins share between 51.7% and 95.6% identity with highly similar protein sizes. NifH is the most conserved protein with 95.6% amino acid identity between the two species (Figure 5B). The sequence of *Enterobacter* sp. R4-368 *nif* operon is deposited to Genbank accession no. KC989924.

### Growth promoting activity of R4-368 is partially attributed to nitrogen-fixation

To elucidate the mechanism of plant growth promotion by *Enterobacter* sp. R4-368, knockout mutants were created for *nifH, nifD* and *nifK* gene individually. The success of gene deletion was confirmed by Southern hybridization (Additional file 2: Figure S1). As expected the *Δnif* mutants were completely devoid of nitrogenase activity in N-free media. There is no significant variation in the endoglucanase activity or in the colony morphology in the *Δnif* mutants. The knockout mutants were able to colonize Jatropha as judged by the high counts of endophytic bacteria population. At 45 DAI, the Wt R4-368-inoculated plants showed an increase of 90%, 64%, 173% and 133% in plant height, leaf number, shoot biomass and root biomass over the mock-inoculated plants, respectively. Interestingly, *nifH, nifD* and *nifK* knockout strains retained substantial growth promoting activity. Consistent with the function of *nif* genes, the leaf nitrogen content of Δ*nifH,* Δ*nifD* and Δ*nifK* inoculated plants were essentially the same as the control whereas it was increased by 118.9% in Wt R4-368-inoculated plants under N-limiting conditions (Table 2). Jatropha seedlings inoculated with Wt and *Δnif* mutants with 2 mM ammonium sulphate significantly increased plant growth parameters, relative chlorophyll content, *in planta* AR-activity and partially recovered N-deficiency in the Δ*nifH,* Δ*nifD* and Δ*nifK* inoculated plants (data not shown).

**Table 2:** Effect of nitrogen fixation genes on growth promotion activity of R4-368 under N-limiting conditions

**Treatments**^**1**^	Plant height (cm)*	Number of leaves*	Shoot biomass (g)*	Root biomass (g)*	**Leaf N content (%)**^**#**^	**N-fixing population (log CFU/g)**^**§**^	***In planta*** **AR activity (nmol C**_**2**_**H**_**4**_**/h/g)**^**§**^
Wild type	34.0 ± 5.8	10.0 ± 2.4	7.1 ± 0.7	1.4 ± 0.1	1.97 ± 0.32	7.47 ± 0.05	49.94 ± 3.18
Δ*nifH*	25.3 ± 4.5^b^	8.1 ± 1.2	4.1 ± 1.3^b^	0.7 ± 0.1	1.06 ± 0.01	5.86 ± 0.10^b^	4.52 ± 2.46^b^
Δ*nifD*	23.1 ± 4.5^b^	7.1 ± 1.2^b^	4.4 ± 0.4^b^	0.6 ± 0.1^b^	0.88 ± 0.02^b^	5.78 ± 0.21^b^	10.47 ± 5.20^b^
Δ*nifK*	22.6 ± 3.9^b^	7.3 ± 1.3^b^	4.4 ± 0.5^b^	0.8 ± 0.1	1.26 ± 0.03	5.69 ± 0.14^b^	8.12 ± 2.55^b^
Mock	17.9 ± 1.3	6.1 ± 1.2^b^	2.6 ± 0.5	0.6 ± 0.0 ^b^	0.90 ± 0.03^b^	5.79 ± 0.15^b^	8.08 ± 2.23^b^

^1^Bacterial inoculants (50 ml/pot) were applied near the root regions after transplanting while the control plant was applied with sterile distilled water. Plants were grown in the open air in a greenhouse. Plants were watered as needed with a nutrient solution containing 5 μM CaCl_2_, 1.25 μM MgSO_4_, 5 μM KCl, 1 μM KH_2_PO_4_, 0.162 μM FeSO_4_, 2.91 nM H_3_BO_3_, 1.14 nM MnSO_4_, 0.76 nM ZnSO_4_, 0.13 nM NaMoO_4_, 0.14 nM NiCl_2_, 0.013 nM CoCl_2_, and 0.19 nM CuSO_4_.

^b^The treatments are not significantly different from each other at 5% threshold.

*, n = 7; #, n = 4; and §, 45 DAI.

### Colonization of *Enterobacter* sp. R4-368 in Jatropha

In order to study the interaction between *Enterobacter* sp. R4-368 and Jatropha at the molecular level, gene transformation and expression systems were developed. Screening of several *E. coli* vectors available in the laboratory for their ability to transform *Enterobacter* sp. R4-368 using the electroporation method identified a highly competent vector, pMC1-EPS-RtGFP (Genbank accession no. KC989925). This vector contains the ColE1 origin of replication of pMK (MrGene, USA), a modified chloramphenicol resistance gene composed of the *E. coli*/bacteriophage T4 *tac* promoter. This drives the expression of *catII* gene derived from pUTmini-Tn5 CM [40], and a modified *eGFP* sequence, Rt*GFP*[41] that is driven by the *eps* gene promoter of *Enterobacter* sp. R4-368.

**Figure 6:**
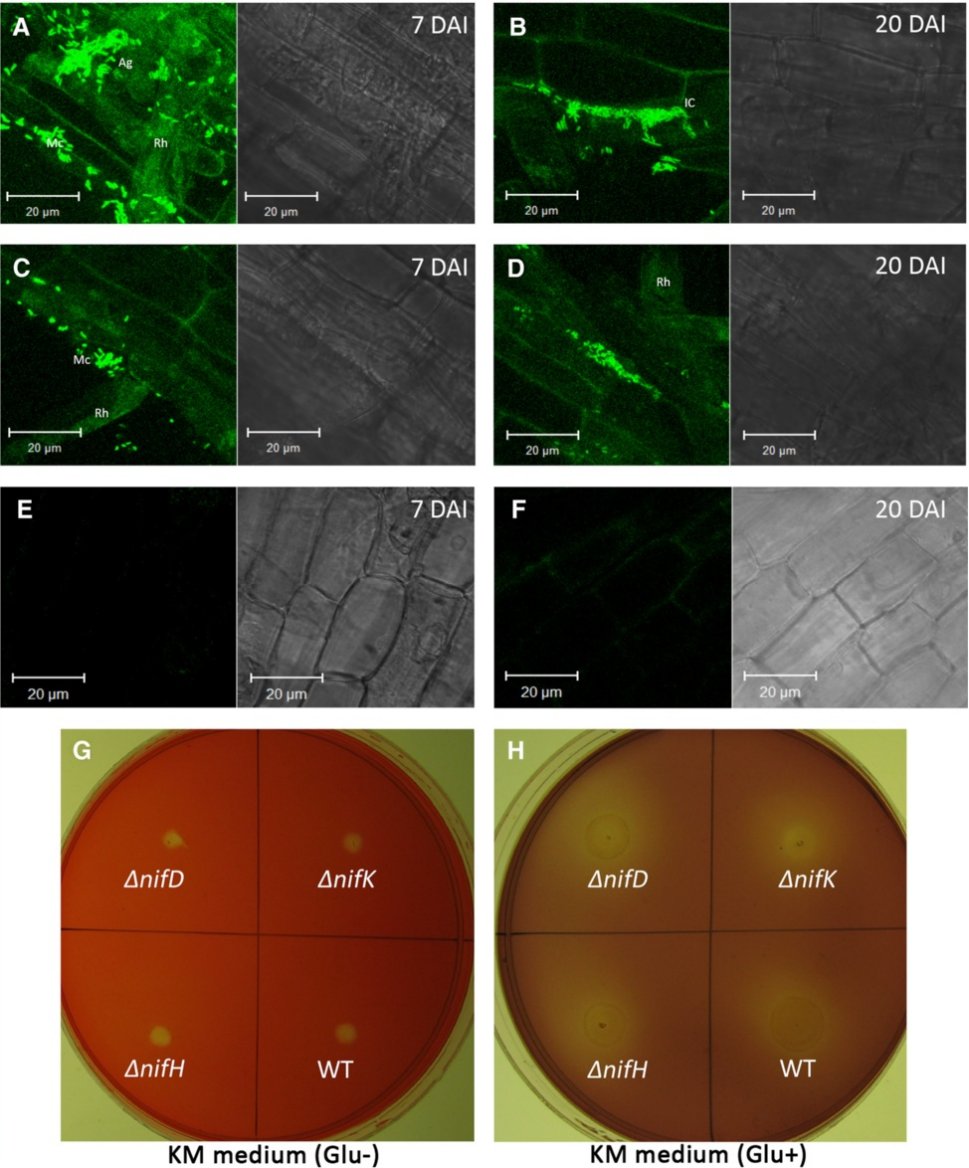
**Plant Colonization and *in vitro *endoglucanase activity of Wt and ∆ *nifK *mutants. (A)** and **(B)**: An optical section of root tissue infected of Wt R4-368 at 7 and 20 DAI respectively. **(C)** and **(D)**: An optical section of root tissue infected of Wt R4-368 *ΔnifH* at 7 and 20 DAI respectively. **(E)** and **(F)** are the mock-inoculated control showing no GFP signal. Scale bars (bar, 20 μm) are shown in each image. Mc: microcolony; Rh: root hair; Ag: cell aggregates; IC: intercellular colonization. **(G)** and **(H)**: Congo-red stained KM agar plate [42] without and with glucose respectively.

To monitor the colonization of *Enterobacter* sp. R4-368 in Jatropha root system, both wild-type and *nif* knockout mutants were transformed with pMC1-EPS-RtGFP, which showed no discernible effect on growth and nitrogenase activity (data not shown). In gnotobiotic assay, GFP tagged cells of both wild-type and *ΔnifH* mutant were observed primarily on the surface and intercellular space of the root tissues (Figure 6A-D). Typically microcolonies were formed on root hairs at 7 DAI and the presence of GFP in intercellular space of the root cortex was observed after 20 DAI (Figure 6D). Similar phenotypes were observed with *ΔnifD* mutants (not shown). Quantification of endophytic bacterial population density in the tissues at 45 DAI revealed small difference between the Wt and *Δnif* mutants. Both strains were also recovered from stem tissues, indicating that they were able to move systemically from root to the upper parts of plants. However, bacteria were not detected in leaf tissues at 45 DAI, suggesting that R4-368 primarily colonized root and stem tissues in jatropha (Table 3).

**Table 3:** Bacterial density at 45 DAI under gnotobiotic conditions

**Treatments**^**1**^	**Endophytic population (log cfu/g)**^**2**^		
	**Root**	**Stem**	**Leaves**
Wild type	7.83 ± 0.10^a^	7.13 ± 0.05^a^	0
*nifH*Δ	7.62 ± 0.16^a^	7.02 ± 0.19^a^	0
*nifD*Δ	7.67 ± 0.20^a^	6.91 ± 0.32^a^	0
*nifK*Δ	7.65 ± 0.14^a^	6.98 ± 0.32^a^	0
Mock	0	0	0

^1^Wt and *nif* mutants were labelled with pMC1-EPS-RtGFP and inoculated to root zones of 12 day-old seedlings derived from surface-sterilized. Plants were maintained in sterile conditions in sand-nutrient mix in Phytatrays.

^2^Number of CFU (per g dry biomass) recovered from surface-sterilized root tissues of *Jatropha curcas* cv. MD-44. Bacteria were recovered on 2xYT medium supplemented with chloramphenical (100 μg/ml).

^a^The treatments are not significantly different from each other at 5% threshold.

## Discussion

Certain definitive evidence that a particular bacterium provides fixed nitrogen to the plant should be provided according to Iniguez et al. [35]. One of the critical evidence is that the bacterial inoculation should increase the nitrogen concentration of plants and relieve the nitrogen deficiency symptoms under nitrogen-limiting conditions. These effects should not be present while using *nif* mutant or uninoculated controls [35]. We tried to illustrate this by inoculating with the wild-type and *Δnif* mutants of our strain in pot cultures under nitrogen-limiting conditions. Besides improving the plant growth and biomass of Jatropha, the wild-type strain significantly increased the content of nitrogen and chlorophyll content of the leaves whereas plants inoculated with *Δnif* mutants showed reduced growth and chlorotic leaves, similar to the non-inoculated control (Table 1 and Table 2).

Increasing reports of natural endophytic bacteria with plant growth promotion (PGP) traits have been reported. Besides nitrogen-fixation, PGP bacteria may modulate plant shoot and root development by secretion of plant hormones or enzymes; such as auxin and 1-aminocyclopropane-1-carboxylate deaminase that reduces ethylene level in plant tissues; enhancement of resistance to pathogens through releasing volatile compounds; solubilisation of soil phosphorus and trace elements [19,21,43]. To date, a small number of endogenous bacteria species have been isolated from *J. curcas*, including *Pseudonocardia sichuanensis*[44], *Nocardia endophytica*[45], *Pleomorphomonas diazotrophica*[46], *Aureimonas jatrophae* and *Aureimonas phyllosphaerae*[47]*, Jatrophihabitans endophyticus*[48] and *Enterobacter cancerogenus*[49]. However, none has been studied on the mechanistic aspects of plant growth promotion in this promising biofuel plant.

In this study, we surveyed the microbial diversity of Jatropha that were cultivated in the tropical region (Singapore) and focused on the *Enterobacter* species that are native to Jatropha. Although a species identified as closely related to *E. cancerogenus* was reported to improve early growth parameters [49], our isolates appeared to be phylogenetically distinct as one group, represented by strains R4-368, shares 98.0% 16S rDNA sequence identity to *E. cancerogenus;* while the second group containing 7 isolates share 97.7-97.8% 16S rDNA sequence identity to that of *E. cancerogenus* (Figure 1). The isolates in second group are more closely related to *E. radicincitans* D5-23^T^, *E. oryzae* Ola51^T^ and *Enterobacter arachidis* Ah-143^T^, which are known PGP bacteria in other crops [50-52].

From the agronomical point of view, *Enterobacter* sp. R4-368 is a promising biofertilizer strain. Apart from the confirmation of early growth parameters (Figures 3 &4, Table 1 & Table 2), which are similar to other findings with a related strain, *E. cancerogenus*[49], we have provided strong evidence that *Enterobacter* sp. R4-368 significantly improved yield parameters even in the presence of nitrogen fertilizer application and competing nitrogen-fixing bacteria that are diverse and abundant in the non-sterilized soil (Figure 4; Additional file 1: Table S1). In addition, we have demonstrated that PGP activity of R4-368 is only partially attributed to its nitrogen-fixing activity as ∆*nif* mutants retained substantial PGP activity (Table 2). Furthermore, we have showed that the *nif* genes of *Enterobacter,* from the result of partial genome sequence of *Enterobacter* sp. R4-368, showed the highest similarity to those of *K. pneumoniae,* which belongs to the same *Enterobacteriaceae* family, and have identical gene organization (Figure 5). In contrast, *Enterobacter* sp. 618, which is a documented efficient PGP bacterium on poplar, lacks the *nif* genes and, hence, growth promotion is believed to be result from secretion of auxin and volatile compounds. Although growth promotion by *Enterobacter* sp. 618 is also associated with increased leaf area, photosynthesis efficiency is not affected by the bacteria inoculation [42].

Colonization of Jatropha roots by strain R4-368 was very similar to that of other endophytes reported so far. Colonization events by diazotrophs have been studied in several grass species. For example, *Azospirillum brasilense* forms a characteristic pattern of wheat colonization that involves invasion of root hair cells [53] and colonization of the root tip [54]. *Herbaspirillum seropedicae* and *Azoarcus* sp. colonized the root cortex, root cap, epidermis, exodermis, and xylem of the host crops studied [55-57]. *Pantoea agglomerans* has been found in the intercellular spaces of wheat roots [58]. The colonization of maize by *K. pneumoniae* was distinct in that it was typically found in the zone of root hair formation but only occasionally formed colonies on or in the root hairs. The pattern of stem colonization contrasted with root colonization whereby the intercellular spaces of the stem cortex were inhabited by single cell or cluster of cells [59]. *Enterobacter cloacae* strain GS1 colonizes rice root under hydroponic conditions showed multicellular aggregates embedded in an extracellular matrix cemented to the root surface on 7 DAI [60]. We found that strain R4-368 formed microcolonies on the root hairs and also in the inter-cellular spaces of the root cortex (Figure 6A-D). When attached to roots, bacteria often increase in numbers by several cell divisions, resulting in the establishment of a microcolony. Invasion of root tissue might take place from such established microcolonies as it was able to secret endoglucanase under *in vitro* conditions (Figure 6G-H). Enumeration of surface-sterilized root/shoots suggests that the bacterium colonized the interior of the tissues since the experimental conditions assured complete sterilization. Another advantage of R4-368 is its ability to move stem tissue, potentially enhancing its nitrogen-fixing capacity *in planta*. In addition, we found that *nif* mutation did not significantly affect colonization of *Enterobacter* sp. strain R4-368. This is similar to the observation with *Acetobacter diazotrophicus* in sugarcane [61] but it is in contrast to *Azoarcus* spp. in which the *nif* mutant lost the ability to colonize and persist in the rhizosphere of kallar grass [56]. It is interesting to note that R4-368 was not detected in leaf tissues. A direction for future research should address if synergism exists when R4-368 is co-inoculated with other plant growth promoting bacteria, e.g., leaf-colonizing nitrogen-fixing strains and mycorrhiza.

## Conclusions

*Enterobacter* sp. strain R4-368 is a very promising biofertilizer inoculant for Jatropha as it not only improved biomass accumulation but also seed yield. Mechanistically, the strains appeared to exert its growth promotion mainly by its ability to fix nitrogen in the rhizosphere and stems, thereby improving nutrient conditions of the plants, which leads to improved photosynthesis due to greater photosynthetic area and chlorophyll content. However, the strain has the other PGP activity that remains to be determined. The application of the strains is likely to significantly improve the commercial viability of Jatropha plantations due to the reduced fertilizer cost and improved oil yield.

## Materials and methods

### Jatropha seedlings

Seeds of *J. curcas* L. cv. MD44 were used throughout the experiments. Surface sterilization of seeds was done by washing coat-less seed kernels in 75% ethanol (v/v) for 1 min and 10% H_2_O_2_ (v/v) for 60 min followed by 3–5 rinses in sterilized distilled water. After soaking overnight at 28°C in darkness, they were germinated on a hormone-free seed germination medium (1/2 MS salt, B5 vitamins, 5 g l^-1^ sucrose, 0.5 g l^-1^ MES and 2.2 g l^-1^ phytagel, pH 5.6) in Phytatrays (Sigma, USA) in a tissue culture room with a temperature of 25°C ± 2°C and 16/8 h light–dark cycles.

### Isolation of endophytic bacteria from Jatropha cultivars

*Jatropha curcas* L. accessions from Indonesia, China and India origins were sampled from the research plots of Agrotechnology Experimental Station located at Lim Chu Kang, Singapore. Root samples with adhering soil were carefully removed with a trowel paper and collected in sterile plastic bags in triplicates. After thorough washings in distilled water, samples were surface-sterilized by washing for 1 min in 90% ethanol and 10 min in 15% H_2_O_2_ followed by 3–5 rinses in sterile distilled water. Tissues were macerated separately in 10 ml 1× PBS using a blender under sterile conditions. A 100-μl sample of the water from the third rinse was plated on rich medium to verify the efficiency of sterilization. Serial dilutions were made, and 100-μl samples were spread -plated on different media [62-64] and incubated at 30°C for up to 7 days for the isolation of endophytes.

### Taxonomical analyses of isolated strains

Genomic DNAs were prepared using the method described by Wilson [65]. PCR amplification of the 16S rRNA genes was carried out with the universal primers 27 F and 1492R [66]. Sequencing was performed using an automated DNA sequencer model 3730 XL (AB Applied Biosystems, HITACHI). Sequence similarity was analyzed against the EzTaxon-e Database (http://eztaxon-e.ezbiocloud.net/) [67] and aligned using CLUSTAL W tool in MEGA version 5.05 [68]. Phylogenetic analyses were performed by the Neighbour-Joining [69], Maximum-Likelihood [70] and Maximum-Parsimony [71] methods using the MEGA version 5.05 [69] with the bootstrap values set at 1000 replications [72].

### Acetylene reduction (AR) assay

Nitrogenase activity in pure cultures was determined by growing strains in a 125 ml serum bottle (Wheaton Industries Inc., USA) in 40 ml nitrogen-free medium (DSMZ medium no. 3), which contains 5.0 g l^-1^ glucose, 5.0 g l^-1^ mannitol, 0.1 g l^-1^ CaCl_2_.2H_2_O, 0.1 g l^-1^ MgSO_4_.7H_2_O, 5.0 mg l^-1^ Na_2_MoO_4_.2H_2_O, 0.9 g l^-1^ K_2_HPO_4_, 0.1 g l^-1^ KH_2_PO_4_, 0.01 g l^-1^ FeSO_4_.7H_2_O, 5.0 g l^-1^ CaCO_3_ and 1 ml trace element mixture. The trace element mixture (SL-6, in DSMZ medium no. 27) is composed of 0.1 g l^-1^ ZnSO_4_.7H_2_O, 0.03 g l^-1^ MnCl_2_.4H_2_O, 0.3 g l^-1^ H_3_BO_3_, 0.2 g l^-1^ CoCl_2_.6H_2_O, 0.01 g l^-1^ CuCl_2_.2H_2_O and 0.02 g l^-1^ NiCl_2_.6H_2_O in water [73]. Acetylene reduction assay was performed by injecting purified acetylene into the bottles sealed with gas-tight serum stoppers to yield 15% acetylene (v/v); this was followed by incubation for up to 96 h at 30°C. To determine the *in planta* nitrogenase activity, AR assay was performed with plants inoculated with isolated strains at 30 and 45 DAI. Root samples were separated from seedlings after carefully removing the adhered soil; placed in 250 ml glass bottles and sealed with a rubber septum. After removing an equivalent volume of air, acetylene was injected into these bottles to give a final concentration of 10% and incubated at 30°C for 24 h. Gas samples (0.5 ml for pure cultures and 0.8 ml for plants) were removed at regular intervals with a PTFE-syringe (Hewlett-Packard, USA) and analyzed in a GCMS-QP2010 Ultra Gas Chromatograph (Shimadzu Corporation, Japan) with an flame ionization detector and GS-Alumina (30 m × 0.53 mm I.D.) column and operated under the following conditions: carrier gas: He-30 ml/min; detector temperature: 200°C; pressure: 4.0 psi. Ethylene produced by the bacteria was quantified using standard ethylene (C_2_H_4_, Product Number: 00489, Sigma-Aldrich) curve prepared in duplicates in concentrations ranging from 1–1000 nmol. All values expressed were obtained after deducting the ethylene values for a blank treatment without samples. For *in planta* AR activity, the values were subtracted against a basal ethylene level which is released by plant tissues, i.e., value of a control with no injected acetylene gas. The protein concentration was determined by a modified Lowry method with bovine serum albumin as standard.

### Molecular techniques and DNA manipulations

DNA isolations, transformations, electrophoration, restriction enzyme digestions, electrophoresis, ligations and hybridizations were performed using standard procedures [65,74]. Electrophoration was carried out with a Micropulser™ (Bio-Rad, Hercules, CA) set at 2.5 kV (12.5 kV cm^-1^); 25 μF; 200 Ω and Gene Pulser® Cuvettes (0.1 cm electrode gap). *Enterobacter* sp. R4-368 was made electrocompetent after overnight growth in 2xYT broth medium at 30°C with shaking (200 rpm). One milliliter of the overnight culture was used to inoculate 500 ml of 2xYT and incubated aerobically at 30°C, with shaking until it reached an *A*_600_ of 0.6 to 0.75, at which point the culture was chilled on ice for 15 min. Bacterial cells were pelleted at 8,000 rpm for 10 min, washed 2 times, first in 75 ml of ice-cold 10% glycerol and then in 50 ml of ice-cold 10% glycerol, and resuspended in 1 ml of ice-cold 10% glycerol. The resultant electrocompetent cells had a transformation efficiency of 10^7^ to 10^9^ CFU/μg of DNA.

For construction of deletion mutant of *nif* genes, approximately 1 kb fragment of the upstream and downstream flanking sequences of the respective gene were amplified by PCR using primers containing appropriate recognition sites for restriction enzymes (Additional file 3: Figure S2). The sequences and details of the primers are mentioned in Additional file 4: Table S2. The amplified PCR fragments were cloned sequentially into the pSTn5-KM (Genbank accession no. KC989926). After DNA sequence verification, deletion cassettes were amplified using primers P1 and P4 (Additional file 4: Table S2); purified with PCR Clean kit (Qiagen, USA) and concentrated by ethanol precipitation. The purified DNA was transformed to *Enterobacter* sp. R4-368 by electroporation. The transformants were confirmed by locus-specific PCR using specific primer targeting a region immediately upstream of downstream of the homology region and a primer targeting the STn5 region. Candidate knockout strains were confirmed by Southern blotting, which was conducted by blotting 1 μg of restriction enzyme-digested genomic DNA onto Hybond N^+^ membranes (GE Healthcare, USA). The blot was hybridized against Dig-labelled DNA probe using DIG High Prime DNA labelling and the targeted DNA was detected according to the Dig starter kit according to the instruction of the manufacturer (Roche Applied Science, Germany). GFP tagging of wild-type and *Δnif* mutants of *Enterobacter* sp. R4-368 was done by electroporation of pMC1-EPS-RtGFP to the competent cells (Genbank accession no. KC989925). Transformants were selected on ABM agar medium with 100 μg ml^-1^ chloramphenicol.

### Bacterial inoculation of Jatropha

*Enterobacter* strains were cultured in 2xYT broth to until exponential growth phase and harvested by centrifugation. After washing once with water, inoculants were made by re-suspending the pellets in water to an OD_600nm_ of 1.2 (~10^8^ cfu per ml). Depending on the experiments, inoculation was done either by seed soaking or watering to the soil. For gnotobiotic assay for root colonization, seedlings were transferred to phytatrays containing 200 g of sterilized sand filled with 40 ml of plant nutrient solution [35]. The phytatrays were covered with lid, and incubated in a growth chamber with a temperature of 28°C and 16/8 h day-night cycles. Bacterial inoculants (5 ml/tray) were diluted into nutrient solution and applied to sand near root zone.

For assessing the impact on seed germination and early growth of the seedlings, seeds were sown in germination trays filled with the pot mixture and inoculated with 2 ml of the inoculants. Root application was done after germination and the growth parameters were observed at 45 DAS. For pot cultivation experiments under N-limiting conditions, healthy seedlings derived from surface-sterilized seeds were transferred to pots containing a mixture of perlite, vermiculite and sand in 1:1:1 ratio (v/v). Bacterial inoculants (50 ml/pot) were applied to the soil around the stems or disturb the top soil before application of inoculants for wetting the root zone. Plants were grown under glasshouse conditions. Plants were watered as needed with a plant nutrient solution containing with 2 mM N or without N-source.

To assess the effects of bacterial inoculation on the growth and yield of Jatropha under natural conditions, two pot culture experiments were conducted with garden soil. Healthy seedlings were transferred from germination trays to the pots and bacterial inoculant (50 ml/pot) were applied near the root zone. Commercial NPK fertilizer was applied about once every 15 days. Biometric observations were recorded once in 30 days. After flowering, yield parameters were recorded once in 30 days.

### Quantification of nitrogen content and leaf chlorophyll

Leaf chlorophyll concentrations were measured using the at LEAF chlorophyll meter (FT Green LLC, Wilmington, USA). Relative chlorophyll concentration is the ratio of transmittance between red (650 nm) and infrared (940 nm) emissions through the leaf. Leaf nitrogen (N) content was determined by the combustion method in an elemental analyzer (*Vario EL Elemental Analyzer*, Elementar, Germany) equipped with a thermal conductivity detector on an Elementar Vario Micro Cube. Accurately-weighed 5-mg ground dried leaf samples were placed in tin capsules for combustion at 1,150°C in the elemental analyzer reactor.

### Confocal laser scanning microscopy (CLSM)

Transverse sections of surface sterilized tissues were cut manually and observed under a CLSM (Zl with motorized stage; Carl Zeiss Inc., Jena GmbH, Germany) equipped with a krypton and an argon laser and supported by the Laser Scanning System LSM5 PASCAL software (Carl Zeiss Inc.). Stacks were obtained using a Microscope Zeiss Axio Imager and the data was analyzed with Zeiss LSM Image Browser version 4.0 program (Carl Zeiss Inc.). Bacterial colonization of Jatropha roots was analyzed from 3-D confocal data stacks.

### Statistical analysis

Bacterial population data were log transformed before being subjected to further analysis. The data were subjected to analysis of variance and testing of means by Duncan’s Multiple Range Test (DMRT) at *P ≤ 0.05* using SAS package, Version 9.2 (SAS Institute Inc., Cary, NC, USA). Student’s t-test was done using the JavaScript maintained by Professor Hossein Arsham, Johns Hopkins Carey Business School, (http://home.ubalt.edu/ntsbarsh/Business-stat/otherapplets/MeanTest.htm).

## Abbreviations

ARA: Acetylene reduction activity; CDS: Coding DNA sequence; CFU: Colony forming units; CLSM: Confocal laser scanning microscopy; DAI: Days after inoculation; DMRT: Duncan’s multiple range test; PGP: plant growth promotion; RAST: Rapid annotation using subsystem technology; RG: Rate of germination; SVI: Seedling vigour index.

## Competing interests

The authors declare that they have no competing interests.

## Authors’ contributions

MM, JL and HY conceived experiments and drafted the manuscript. MM performed strain isolation and characterization and bioassays for bacteria and plants. PN and CHI prepared DNA constructs for gene knockout and GFP tagging. NST participated in the PGP traits experiments and data analysis. CL, FL, CR participated in the bioassay for plants. All the authors have read and approved the final manuscript.

## Authors’ information

JL is Head of the laboratory and a Director of the Institute’s Strategic Research Program. MM is a postdoctoral fellow, PN is PhD student, CL is senior Research Officer, and FL, CHI and NST are Assistant Research Officer at the Temasek Life Sciences Laboratory, 1 Research Link, NUS, Singapore 117604. HY is Chief Scientific Officer and CR is Manager (Agronomist), Joil Pte Ltd, 1 Research Link, National University of Singapore, Singapore 117604.

## Supplementary Material

Additional file 1: Table S1 Effects of R4-368 inoculation on flower sex ratio and seed yield parameters of Jatropha. Click here for file

Additional file 2: Figure S1 Molecular construction and characterization of *Δnif* mutants. **(A)** Acetylene reduction activity compared with strain R4-368 and their nif mutants, **(B)** DNA blot analysis. Genomic DNA (gDNA) was isolated from *ΔnifH,ΔnifD* and *ΔnifK* knock-out mutants. Genomic DNA aliquots (1 μg) were digested with individual restriction enzymes and subjected to agarose gel electrophoresis. The DNA blot was hybridized with a DIG-labeled *nifH, nifD* and *nifK*-flanking region as DNA probe. WT-wild type strain R4-368; M- DNA Molecular Weight Marker III (Roche Applied Science, Germany). Click here for file

Additional file 3: Figure S2 Map showing *ΔnifH ***(A)***, ΔnifD ***(B)** and *ΔnifK ***(C)** knock out constructs. Click here for file

Additional file 4: Table S2 List of primers used in this study. Click here for file
