# Supplementary material for: Improvement of plant growth and seed yield in Jatropha curcas by a novel nitrogen-fixing root associated Enterobacter species
Source: Biotechnol Biofuels. 2013 Oct 1;6:140. doi: 10.1186/1754-6834-6-140 (PMC3879406; doi:10.1186/1754-6834-6-140)
Supplement: Additional file 1: Table S1 — Effects of R4-368 inoculation on flower sex ratio and seed yield parameters of Jatropha. [file 1754-6834-6-140-S1.doc]

Additional file 1: Table S1 Effects of R4-368 inoculation on flower sex ratio and seed yield parameters of Jatropha.
Treatment	Number of plants treated	Number of inflorescences observed†	Total female flowers	Total male flowers	Ratio female: male flower	Number  of fruits‡	Number of seeds‡	Seed weight (g)‡	
Trail I	 	 	 	 	 	 	 	 	
Control	8	25	42 ± 1.73	630 ± 30.0	1:15	4.75 ± 0.60	13.50 ± 0.58	6.86 ± 0.42	
Treated	8	25	86 ± 3.21	688 ± 14.01	1:8	13.25 ± 1.36	37.50 ± 2.31	19.55 ± 1.56	
Trail II	 	 	 	 	 				
Control	12	50	175 ± 3.51	2750 ±104.08	1:16	10.67 ± 0.67	28.58 ± 1.72	13.56 ± 1.19	
Treated	12	50	425 ± 16.20	2906.3 ±123.0	1:7	15.83 ± 0.94	42.58 ± 1.78	21.79 ± 1.67	
†Number of inflorescences observed from different time intervals. 
‡Number of fruits/plant, number of seeds/plant and seed weight/plant were recorded on 480 and 540 DAI from Trail I and II, respectively.
