# Supplementary figures and images for: Improvement of plant growth and seed yield in Jatropha curcas by a novel nitrogen-fixing root associated Enterobacter species
Source: Biotechnol Biofuels. 2013 Oct 1;6:140. doi: 10.1186/1754-6834-6-140 (PMC3879406; doi:10.1186/1754-6834-6-140)

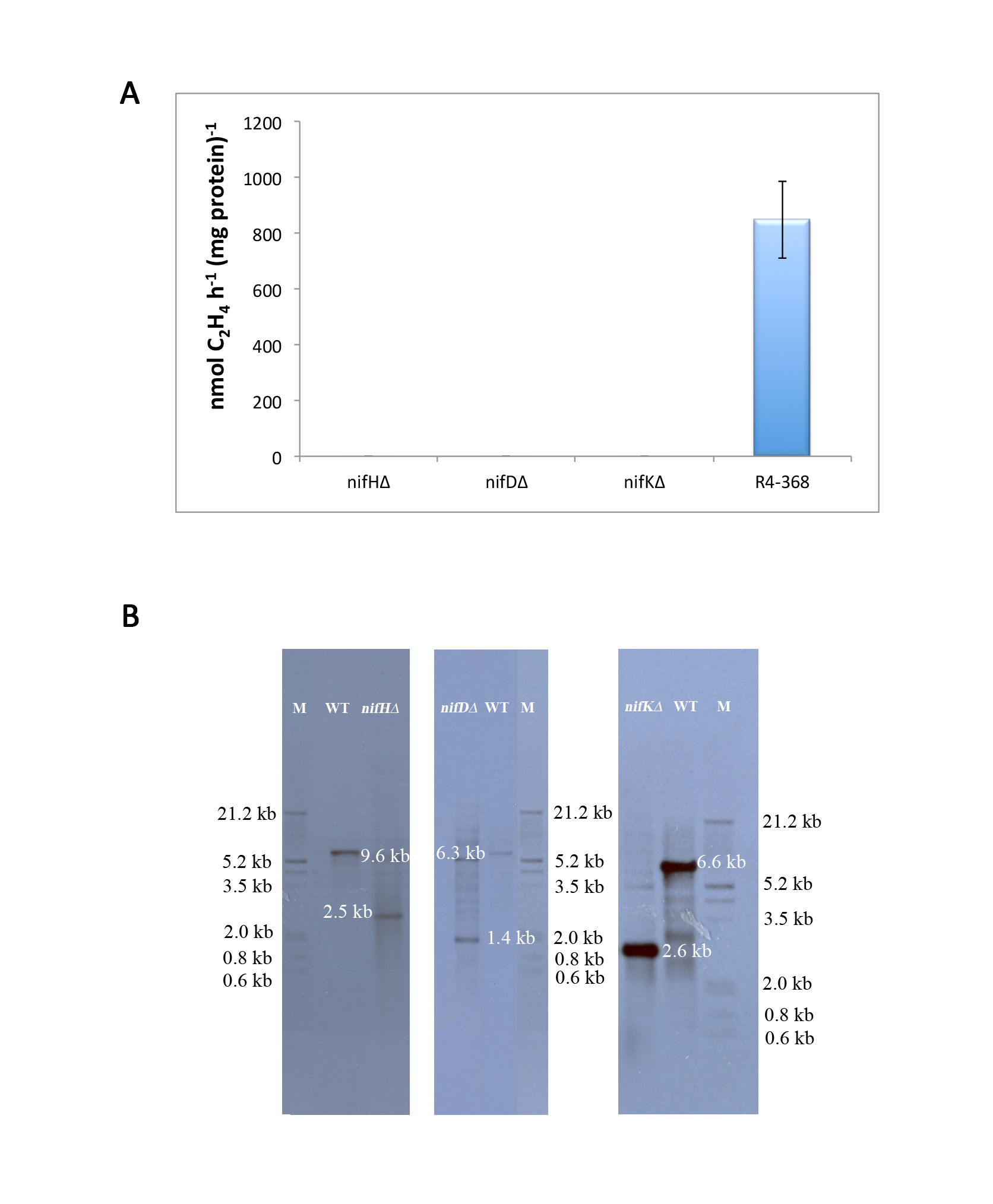

Supplement: Additional file 2: Figure S1 — Molecular construction and characterization of Δnif mutants. (A) Acetylene reduction activity compared with strain R4-368 and their nif mutants, (B) DNA blot analysis. Genomic DNA (gDNA) was isolated from ΔnifH,ΔnifD and ΔnifK knock-out mutants. Genomic DNA aliquots (1 μg) were digested with individual restriction enzymes and subjected to agarose gel electrophoresis. The DNA blot was hybridized with a DIG-labeled nifH, nifD and nifK-flanking region as DNA probe. WT-wild type strain R4-368; M- DNA Molecular Weight Marker III (Roche Applied Science, Germany). [file 1754-6834-6-140-S2.jpeg]
