# Supplementary material for: Improvement of plant growth and seed yield in Jatropha curcas by a novel nitrogen-fixing root associated Enterobacter species
Source: Biotechnol Biofuels. 2013 Oct 1;6:140. doi: 10.1186/1754-6834-6-140 (PMC3879406; doi:10.1186/1754-6834-6-140)
Supplement: Additional file 3: Figure S2 — Map showing ΔnifH (A), ΔnifD (B) and ΔnifK (C) knock out constructs. [file 1754-6834-6-140-S3.ppt]

## Slide 1
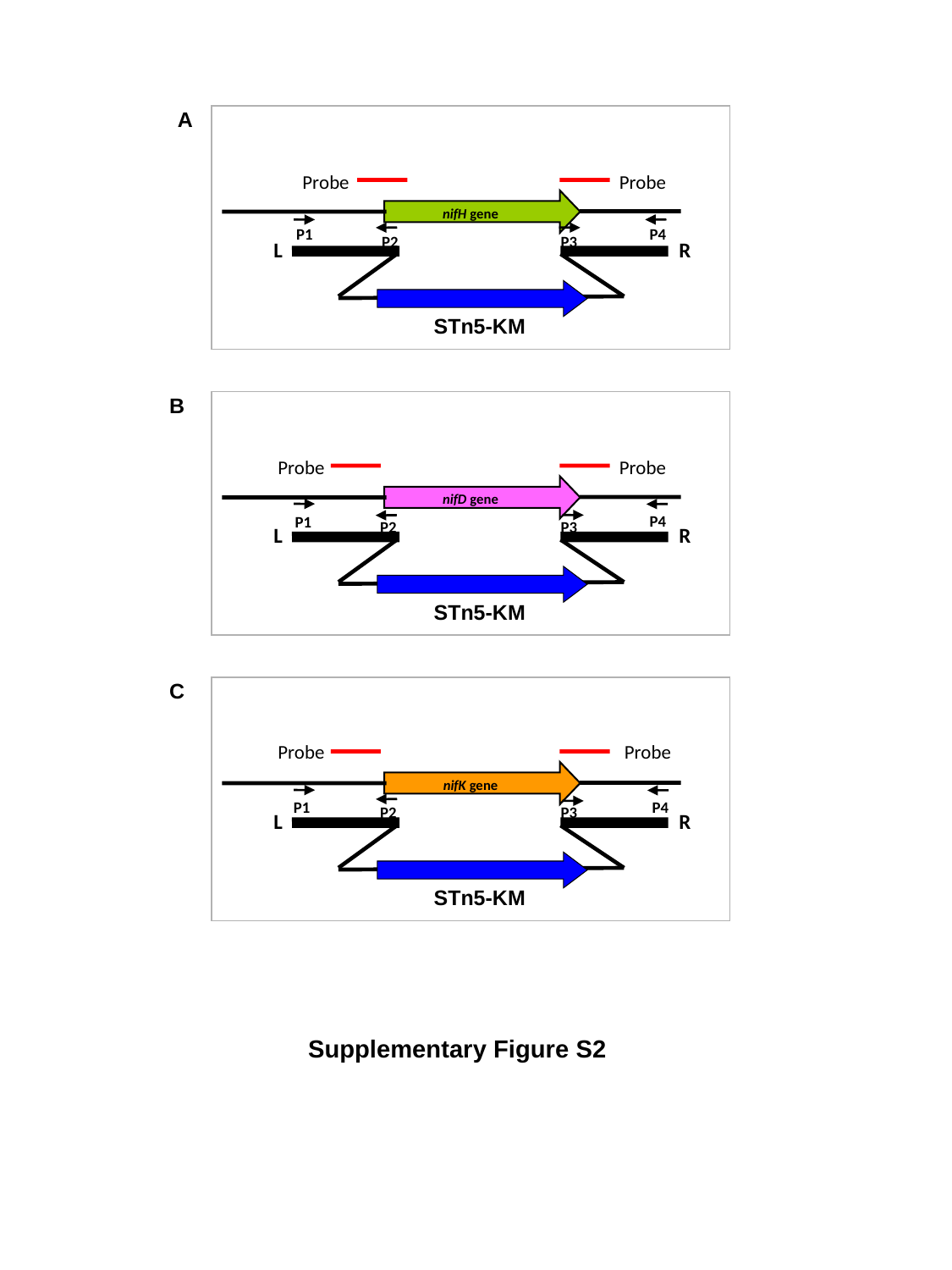

A
Probe
Probe
nifH gene
P1
P4
P2
P3
L
R
STn5-KM
B
Probe
Probe
nifD gene
P4
P1
P2
P3
L
R
STn5-KM
C
Probe
Probe
nifK gene
P1
P4
P3
P2
L
R
STn5-KM
Supplementary Figure S2
