# Supplementary material for: Improvement of plant growth and seed yield in Jatropha curcas by a novel nitrogen-fixing root associated Enterobacter species
Source: Biotechnol Biofuels. 2013 Oct 1;6:140. doi: 10.1186/1754-6834-6-140 (PMC3879406; doi:10.1186/1754-6834-6-140)
Supplement: Additional file 4: Table S2 — List of primers used in this study. [file 1754-6834-6-140-S4.doc]

Additional file 4: Table S2 List of primers used in this study.
Primer name	Sequence (5' to 3')	
nifH-H1-U-SpeI  (P1) 	GACTAGTCGCATGGTCATGGTGTTTCTCC	
nifH-H1-L-XhoI  (P2) 	CCGCTCGAGCGGTTCGCGCGCCTGAAAGTAGATAT	
nifH-H2-U-BamHI  (P3)	CGGGATCCCGTCGTTCTGCGCCTGGTAT	
nifH-H2-L-EcoRV   (P4)	AAAGATATCGGAGTTCGGCATTATGGATGTGGA	
nifD-HI-U-SpeI    (P1)	GACTAGTCCACATCCATAATGCCGAACTCC	
nifD-H1-L-XhoI   (P2)	CCGCTCGAGCGGACGCGACAGCTTGTTGAAC	
nifD-H2-U-BamHI  (P3)	CGGGATCCCGCCTTGCCGGTTAACTGACTGAT	
nifD-H2-L-EcoRV   (P4)	AAAGATATCCCGTTTGTTCACCGATTTGTGG	
nifK-H1-U-SpeI    (P1)	GACTAGTCGGTGAACAAACGGGATAACAGG	
nifK-H1-L-XhoI   (P2)	CCGCTCGAGCGGACCCGGAGCAGGAAA	
nifK-H2-U-BamHI  (P3) 	CGGGATCCCGCAAATCGCAAGCCAGAAAATAGTGA	
nifK-H2-L-EcoRV   (P4) 	AAAGATATCGATCTTATCCGTTAACCATCACAC	
nifH-for 	ACCTGGAATTAGAAGACGTGCTG	
nifH-rev	CAGGTCGGGTCATATTCGATAAC	
nifD-for	CGAACGTAACCTGGAGATAATCG	
nifD-rev	TCTCCATTCCCAGGTCTTCATAG	
nifK-for	ATACGAAGCGCTGAACTTCAAAC	
nifK-rev	TTGTTACCGTTATGGCAGAGGAT	
Note: Restriction enzyme sites are underlined. P1-P4 denotes the position of the primer in Figure S1.
